# DIAPH1-MFN2 interaction decreases the endoplasmic reticulum-mitochondrial distance and promotes cardiac injury following myocardial ischemia

**DOI:** 10.1038/s41467-024-45560-0

**Published:** 2024-02-17

**Authors:** Lorrie A. Kirshenbaum, Rimpy Dhingra, Roberto Bravo-Sagua, Sergio Lavandero

**Affiliations:** 1 The Institute of Cardiovascular Sciences, St. Boniface Hospital Albrechtsen Research Centre, Department of Physiology and Pathophysiology, Winnipeg, Canada; 2 Rady College of Medicine, Max Rady Faculty of Health Sciences, University of Manitoba, Winnipeg, Manitoba R2H 2H6 Canada; 3 Laboratory of Obesity and Metabolism (OMEGA), Institute of Nutrition and Food Technology (INTA), Universidad de Chile, Santiago, Chile; 4 Interuniversity Center for Healthy Aging (CIES), Consortium of Universities of the State of Chile (CUECH), Santiago, Chile; 5 Advanced Center for Chronic Diseases (ACCDiS), Faculty of Chemical & Pharmaceutical Sciences & Faculty of Medicine, University of Chile, Santiago, Chile; 6 Department of Internal Medicine, Cardiology Division, University of Texas Southwestern Medical Center, Dallas, TX USA

**Keywords:** Stress signalling, Calcium signalling

## Abstract

Contact between organelles such as the mitochondria (Mito) and endoplasmic reticulum (ER) is crucial to coordinate vital cellular homeostatic processes. Here we discuss recent work showing that Mito-ER proximity is regulated by heterotypic complexes between the F-actin polymerizing protein Diaphanous-1) and the mitochondrial dynamics protein Mitofusin 2, which confers increased susceptibility to ischemia/reperfusion injury.

In the heart, the mitochondrion is the primary source of ATP through the oxidative metabolism of biofuels such as lipids and carbohydrates. However, the mitochondrion also plays critical roles buffering intracellular Ca2^+^, exchanging lipids and long-chain fatty acids, and ion transport as well as providing a signaling platform for apoptosis and necrosis^1^. The fidelity of these mitochondrial-regulated processes relies on complex interplay with other organelles including the nucleus, peroxisomes, lysosomes, and endoplasmic reticulum (ER). Indeed, there is a growing awareness that intra-organelle contact sites provide a communication hub for organelle-organelle cross-talk important for nutrient sensing, intracellular trafficking, and the exchange of proteins and metabolites. Defects in inter-organelle communication have been postulated as an underlying cause of a variety of human diseases, including cancer, diabetes, neurodegenerative, and cardiovascular diseases^2^.

Inter-organelle communication is critical for efficient metabolic signaling and intracellular trafficking and governed by organelle-organelle proximity, organelle heterogeneity, and intracellular location^3^. Notably, both homotypic interactions between the same organelle and heterotypic interactions between different organelles can occur. For example, homotypic interactions between mitochondria have been well-documented during mitochondrial fusion and fission events in the context of mitochondrial biogenesis and mitophagy.

Conversely, among the best-studied examples of intra-organelle heterotypic interaction involves the mitochondria and ER (Mito-ER), which has been studied in the context of a number of human diseases such as cardiac hypertrophy, diabetes, and neurodegenerative diseases such as Alzheimer’s and Parkinson^4,5^. Mito-ER tethering involves protein bridges between the mitochondrial fusion protein Mitofusin 2 (MFN2), the ER-Mitochondria Encounter Structure (ERMES)^6^ and Mito-PTPIP51 and ER- VAPB^7^. The role of mitochondrial MFN2 in regulating mitochondrial fusion, metabolism, and ER tethering is well-studied^7,8^. Recently, the Scorrano group^9^ identified ER-restricted, alternatively spliced variants of MFN2 that encode functionally different proteins. ERMIT2 predominantly exists at the Mito-ER interface and is involved in Mito-ER tethering, whereas ERMIN2 is localized to non-Mitochondrial Associated Membrane (MAM) regions of the ER and regulates ER morphology. Although Mito-ER tethers and contact sites are critical for maintaining intracellular homeostasis, there is a delicate balance in which either too little or too much Mito-ER apposition can be detrimental, resulting in abnormalities in metabolic signaling, organelle morphology, and nutrient and Ca^2+^ handling that ultimately results in cell death. Clearly, it is critical to dynamically maintain the distance between these organelles^10^. However, little is known about the cellular mechanisms that regulate Mito-ER interactions in the heart under normal and disease conditions. A study by Yepuri et al. ^11^ recently published in *Nature Communications* identifies a new compelling role for Diaphanous-1 (DIAPH1), a formin protein involved in actin F- polymerization, as a key regulator of Mito-ER/SR proximity (Fig. 1).

**Fig. 1:**
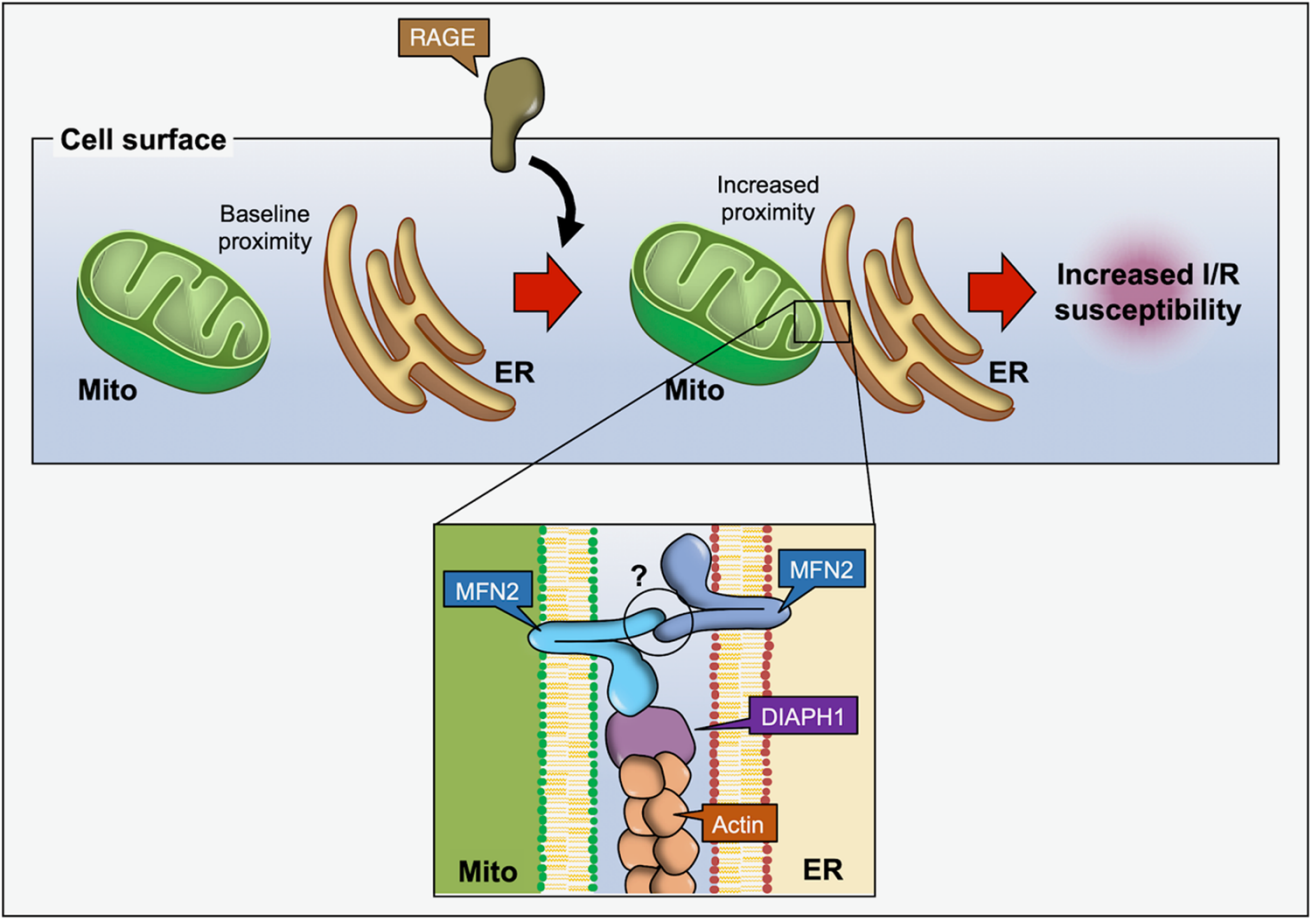
DIAPH1-MFN2 interaction increases Mito-ER proximity, rendering cardiac myocytes susceptible to I/R injury. Activation of the Receptor of advanced glycation end-products (RAGE) promotes the interaction between Diaphanous 1 (DIAPH1), a formin protein involved in actin F- polymerization, and Mitofusin 2 (MFN2), a GTPase that mediates mitochondria (Mito)-endoplasmic reticulum (ER) physical tethering. As a consequence of DIAPH1-MFN2 binding, the Mito-ER distance decreases, which renders cardiac myocytes prone to damage upon ischemia/reperfusion (I/R) injury. However, it remains unknown whether this increased proximity is mediated by homotypic interaction between MFN2 at the surface of both Mito and the ER, or by the interaction of MFN2 with other ligands.

## DIAPH1-MFN2 interactions increase Mito-ER proximity

Using a variety of in vivo and in vitro models, Yepuri et al. demonstrated that during ischemia-reperfusion (I/R), which simulates ischemic heart disease, the interaction between DIAPH1 and MFN2 was markedly increased compared to non-ischemic controls. Previously, this group reported a pathological role for DIAPH1 during ischemic injury;^12^ although the underlying mechanism was not determined. In the present study, the authors discovered that the damaging effects of I/R were directly related to the increased proximity between Mito-ER caused by the increased interaction between DIAPH1 and MFN2, causing abnormal Ca^2+^ handling and metabolic dysfunction. The authors further showed that interactions between DIAPH1 and MFN2 involved the Diaphanous Inhibitory Domain (DID domain) of DIAPH1 and the cytosolic domain of MFN2 containing the GTPase activity as well as being actin polymerization-dependent^11^. Interestingly, DIAPH1 interactions were predominantly in SR region and were limited to MFN2, with the similar mitochondrial fusion protein MFN1 failing to interact.

## Cardiotoxic role of DIAPH1

The role of DIAPH1 in maintaining Mito-ER proximity under basal and hypoxia/reoxygenation (H/R) or I/R was verified in vitro using H9C2 cell line deficient for DIAPH1 as well as in vivo studies using cardiac-restricted DIAPH1 knockout mice. Interestingly, the authors found that the inactivation of DIAPH1 decreased apposition between Mito and ER, thus suppressing ER stress, mitochondrial injury, and cardiac dysfunction. Further, Yepuri et al. next showed that synthetic linkers that bridge Mito-ER interactions could cancel out the protective effects of DIAPH1 inhibition. Together, these findings substantiate that increased proximity of Mito-ER mediated by DIAPH1 promotes cardiac injury following I/R^11^.

Furthermore, Yepuri et al. used different cell types as well as in vivo mouse models to demonstrate a functional link between DIAPH1-MFN2 and mitochondrial senescence. Overall, this work demonstrates time that cardiac dysfunction associated with I/R is obligatorily linked to and mutually dependent upon the Mito-ER proximity dictated by DIAPH1-MFN2 complexes. The study identifies DIAPH1 as potential therapeutic target to modulate cardiac injury following I/R.

## DIAPH1-MFN2 and AGEs

Yepuri et al. also explored the role of advanced glycation end-products (AGEs) in the regulation of Mito-ER proximity. In a previous study, they showed a functional interaction between DIAPH1 and the receptor for advanced glycation end products (RAGE)^13^. In their current work, knockdown of RAGE decreases Mito-ER proximity, while activation with the AGE carboxymethyllysine (CML) results in increased DIAPH1-MNF2 interaction. However, inhibition of RAGE-DIAPH1 binding using a small molecule impairs DIAPH1-MFN2 interaction, thereby highlighting the role of RAGE in regulating Mito-ER physical coupling. Taken together, these results suggest that increased CML in the heart may decrease Mito-ER distance, thus rendering the heart more susceptible to damage upon I/R injury. This is very compelling, because heart CML levels correlate with age, diabetes mellitus, and coronary heart disease^14^, thus providing a physiological mechanism for the well-known observation that hyperglycemia and age are major mortality risk factors after myocardial infarction^15^.

## Exploration of DIAPH1 associations with global MFN2 vs organelle-specific MFN2

Although compelling new evidence has demonstrated a role for DIAPH1-MFN2 protein complexes in regulating Mito-ER apposition under basal and I/R stress, there are several questions that remain unanswered. Because MFN2 is a rather promiscuous protein present in many organelles; it is unknown whether DIAPH1 interacts with MFN2 localized to different organelles or is restricted to only MFN2 proteins localized to the ER. Based on the proximity ligation assay data, the authors concluded that DIAPH1-MFN2 interactions occurs around ER. However, close inspection of the data reveals proximity ligation assay staining puncta in regions of the cell without a positive ER signal, raising the strong possibility that DIAPH1-MFN2 interactions may not be limited to ER but instead may interact with other membranous organelles such as peroxisomes and mitochondria. Testing whether DIAPH1-MFN2 protein-protein complexes can be detected in peroxisomes, mitochondria, and ER would provide definitive proof and broad significance to DIAPH1-MFN2 interactions beyond their ascribed role in calcium regulation and metabolic signaling.

In addition, there are two isoforms of MFN2 that have been identified localized to ER, ERMIT2 and ERMIN2^9^. It remains to be tested whether DIAPH1 interacts with one or both ER MFN2 isoforms and whether specific MFN2 isoforms influence Mito-ER tethering and cardiac function. Although calcium dynamics are controlled by Mito-ER interactions that are critical for normal cardiac function, this potential function of the DIAPH1-MFN2 complex was not fully explored. It is unclear how altered ER-Mito interactions influence cardiac contractility or whether Mito-ER apposition influences ER morphology or ER protein trafficking. Furthermore, future studies should assess sex-specific differences between female and males, as it has been established that sex differences exist with respect to cardiac injury following myocardial infarction, particularly heart failure. It would be important to know whether plasticity in Mito-ER contact sites influences cardiac outcomes following I-R in a sex-specific manner.

Mitochondria in cardiac myocytes are highly heterogeneous organelles with perinuclear, intrafibrillar, and subsarcolemmal pools. Do changes in DIAPH1-MFN2 interactions affect the global mitochondrial network, or are they restricted to a specific compartmental pool of mitochondria? It would also be prudent to assess DIAPH1-MFN2 interactions and Mito-ER contacts in adult cardiac myocytes, which exhibit a different morphology, distribution of organelle, and network from the cell lines used in this study. Another exciting future direction is the interactions identified between DIAPH1 and other Mito-ER-associated proteins, such as voltage-dependent anion-selective channel 1 (VDAC1) or Inositol trisphosphate receptor (IP3R). The relevance of these DIAPH1 interactors to Mito-ER proximity and cardiac myocyte function remains to be investigated.

## Concluding remarks

Yepuri et al. discovered a previously unknown association between cytoplasmic F-actin polymerizing protein DIAPH1 and the mitochondrial dynamics protein MFN2 that influences cardiac function by controlling the distance at the Mito-ER contact site. Hence, the interaction between DIAPH1 and MFN2 exemplifies how distinct cellular compartments communicate to evoke an integrated response to cell stress. It will be important in future studies to assess the role of DIAPH1 as a modulator of intra-organelle communication in the pathogenesis of human diseases, particularly where altered MFN2 activity is known to play a role.
